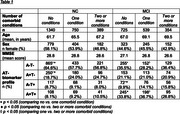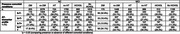# Associations of multiple comorbidities and Amyloid and Tau Biomarker Profiles

**DOI:** 10.1002/alz70856_099639

**Published:** 2025-12-24

**Authors:** Stephan Duijkers, Inez H.G.B. Ramakers, Kim Massloh, Veerle van Gils, Jolien van der Velden, Julie E. Oomens, Stephanie J. B. Vos, Pieter Jelle Visser, Willemijn J. Jansen, the Amyloid Biomarker Study group

**Affiliations:** ^1^ Alzheimer Center Limburg, School for Mental Health and Neuroscience, Maastricht University, Maastricht, Limburg, Netherlands; ^2^ Maastricht Universy Medical Centre, department of Psychiatry and Psychology, Maastricht, Limburg, Netherlands; ^3^ Wisconsin Alzheimer's Disease Research Center, University of Wisconsin School of Medicine and Public Health, Madison, WI, USA; ^4^ Alzheimer Center and Department of Neurology, Amsterdam Neuroscience Campus, VU University Medical Center, Amsterdam, Netherlands; ^5^ Alzheimer Center Limburg, School for Mental Health and Neuroscience, Maastricht University, Maastricht, Netherlands

## Abstract

**Background:**

With an aging global population, incidence of Alzheimer's disease (AD) and age‐related comorbid conditions are increasing. Hypertension (HT), diabetes mellitus (DM) and hypercholesterolemia (HCHOL) are related to dementia, it remains unclear if (a combination of) these are associated with AD pathology. Our current study investigates associations between combinations of HT, DM and HCHOL and amyloid‐β‐42 and *p*‐tau pathology in cerebrospinal fluid.

**Method:**

2479 participants with normal cognition (NC) and 1618 participants with Mild Cognitive Impairment (MCI) from 41 studies with data on presence of HT, DM or HCHOL were included from the Amyloid Biomarker Study. Four AT‐biomarker profiles were created, with normal or abnormal amyloid‐β‐42 (A) or phosphorylated tau (T) (using data‐driven cut‐offs). Participants were classified as having no, one, or two or more comorbidities. We compared frequencies of AT profiles across comorbidity groups and presence of separate comorbidities with independent samples t‐tests.

**Result:**

In the NC group, those with one or two or more comorbidities more often were A+T‐ (Table 1, *p* = 0.005 and *p* =  0.014), and less often A‐T‐ (*p* = 0.002; *p* =  0.005), compared to no comorbidities. Presence of HT and HCHOL were related to higher frequency of A+T‐ (Table 2, *p* = 0.003; *p* =  0.014), only presence of HT was related to lower frequency A‐T‐ (*p* = 0.001). In the MCI group, those with one or two or more comorbidities more often were A‐T+ (*p* = 0.023; *p* =  0.005), those with one comorbidity were less frequently A‐T‐ (*p* = 0.008, *p* =  0.011) and those with 2 or more comorbidities were less frequently A+T+ (*p* = 0.018; *p* = 0.002). Presence of HT and HCHOL were related to higher A‐T+ (*p* = 0.01; *p* =  0.027), presence of HCHOL was related to lower A+T+ (*p* = 0.003).

**Conclusion:**

In NC, presence of comorbidities is related to A+T‐, while in MCI, comorbidities were associated to A‐T+ and reversely to A+T+. HT and HCHOL but not DM were differentially related to AT profiles. The current results may have implications for diagnostics in memory clinics.